# LDAR outperforms other albumin-derived indices in predicting 28-day ICU mortality in critically ill myocardial infarction patients: a two-cohort study

**DOI:** 10.3389/fmed.2026.1801925

**Published:** 2026-05-15

**Authors:** Xiongwei Meng, Yi Ou, Jialin Mao, Hongsheng Liao, Junhong Wu, Lin Zhang, Xingkai Qian, Siyuan Yang

**Affiliations:** 1 Department of Cardiac Surgery, Affiliated Hospital of Guizhou Medical University, Guiyang, China; 2 Center for Translational Medicine, Guizhou Medical University, Guiyang, China

**Keywords:** acute myocardial infarction, albumin, correlation analysis, inflammatory nutrition composite index, risk prediction

## Abstract

**Background:**

Early risk stratification is crucial for improving outcomes in critically ill patients with acute myocardial infarction (AMI). Albumin-derived composite indices hold promise as convenient and effective predictive tools, but their relative efficacy and clinical utility remain unclear.

**Methods:**

This two-cohort retrospective analysis utilized a derivation cohort from the MIMIC-IV public database and an external validation cohort from the ICU of Guizhou Medical University Affiliated Hospital. Six albumin-derived composite indices were evaluated. Statistical analyses employed Cox proportional hazards regression models to assess their association with mortality. Predictive performance was compared using the area under the receiver operating characteristic curve (AUC) and Delong’s test. A multivariate risk prediction model was developed based on key prognostic variables selected by multiple machine learning algorithms.

**Results:**

The study included 4,850 critically ill AMI patients (4,210 in the derivation cohort, 640 in the validation cohort). Multivariable-adjusted analysis identified the red cell distribution width to Albumin Ratio (RAR), Urea nitrogen to Albumin Ratio (UAR), and Lactate Dehydrogenase to Albumin Ratio (LDAR) as independent predictors of 28-day ICU mortality. Among these, LDAR demonstrated the strongest predictive ability, with an AUC of 0.702 in the derivation cohort, a finding robustly validated externally (AUC = 0.703). Subgroup analysis indicated consistent predictive value across most populations but revealed a significant interaction with hyperlipidemia. Incorporating LDAR into traditional critical illness scores (e.g., APACHE II, SOFA) significantly improved their predictive discrimination (all Delong’s test *p* < 0.05). A comprehensive model integrating 7 key variables (including LDAR, urea nitrogen, and lactate) selected by machine learning showed good and robust discriminative performance in both internal and external validation (AUCs of 0.767 and 0.735, respectively), significantly outperforming five traditional risk scores (all Delong’s test *p* < 0.05).

**Conclusion:**

Among the six albumin-derived composite indices, LDAR offers the best independent and incremental predictive value for 28-day ICU mortality in critically ill AMI patients. Its interaction with hyperlipidemia suggests potential for targeted risk stratification. The machine learning model incorporating LDAR and other variables demonstrates robust performance, providing a promising tool for the early clinical identification of high-risk patients.

## Background

Cardiovascular disease is the leading cause of death worldwide, with acute myocardial infarction (AMI) representing a critical event associated with high mortality (1, 2). Despite significant advances in reperfusion and pharmacological therapies, which have contributed to an overall decline in AMI mortality, the prognosis for critically ill patients—particularly those admitted to the ICU, remains poor. For instance, cardiogenic shock following AMI still carries a 28-day mortality rate of approximately 40% (3, 4). The pathological core of AMI involves irreversible myocardial cell necrosis due to acute coronary occlusion, triggering a severe inflammatory response and ventricular remodeling (5, 6). In this context, the early and accurate identification of high-risk patients is crucial for improving outcomes. However, existing clinical prediction tools are often limited in practical use due to their complexity and inconvenience (7–9). Consequently, the search for and validation of novel biomarkers or composite indices that are both convenient and effective for predicting outcomes in critically ill AMI patients has become an important focus of clinical research.

Serum albumin, the most abundant circulating protein in the human body, plays key physiological roles in maintaining colloid osmotic pressure and exerting anti-inflammatory and antioxidant effects. It is therefore widely used as an important biomarker for assessing nutritional status and systemic inflammation (10–12). Clinical studies have consistently shown that hypoalbuminemia is associated with adverse outcomes in various conditions, including heart failure, acute pulmonary embolism, diabetic nephropathy, and ICU stays (13–15). However, as a single indicator, serum albumin levels can be influenced by numerous factors such as liver function, hydration status, acute stress response, and clinical interventions (e.g., fluid administration, nutritional support), which limits its predictive stability and specificity (13–15). To overcome the limitations of a single marker, recent research has focused on combining albumin with other complementary laboratory parameters that reflect related pathophysiological processes. These include red cell distribution width (RDW), which indicates red blood cell heterogeneity; lactate, representing tissue perfusion and metabolic state; anion gap, reflecting acid–base balance; and lactate dehydrogenase (LDH), suggesting cellular injury (16–21). Composite scores integrating such markers can provide a more comprehensive profile of a patient’s inflammatory, nutritional, and metabolic stress state. Such indices have demonstrated superior predictive performance compared to albumin alone for prognosis in various critical conditions, including heart failure, myocardial infarction, and sepsis (16–21).

Nevertheless, the existing evidence is largely derived from retrospective data, and there is a lack of direct, systematic comparison among the various albumin-derived composite indices. Their relative predictive merits and optimal clinical application scenarios remain unclear. Given the substantial resource and time investments required for prospective cohort studies, it is imperative to first use retrospective data to identify which integrated index offers the best combination of simplicity and predictive power. This approach can prioritize candidates for subsequent high-quality research and optimize resource allocation. Therefore, this study aims to systematically evaluate and compare the performance of several albumin-derived inflammatory-nutritional composite indices in predicting 28-day ICU mortality among critically ill myocardial infarction patients, with the goal of identifying the optimal predictive tool.

## Methods

### Data sources

This study utilized two retrospective cohorts. The derivation cohort was sourced from the publicly available Medical Information Mart for Intensive Care IV (MIMIC-IV) database, version 2.2 (22). This database contains information for 196,527 adults admitted to the Beth Israel Deaconess Medical Center between 2008 and 2019. Database access was granted under an approved protocol. The Institutional Review Boards of the Massachusetts Institute of Technology approved the use of the MIMIC-IV database for research and waived the requirement for informed consent due to the retrospective nature of the study and the use of de-identified data. The external validation cohort included AMI patients treated in the Intensive Care Unit of Guizhou Medical University Affiliated Hospital. Following approval from the hospital’s Ethics Committee, this cohort was enrolled between January 2015 and January 2025. The Committee waived the requirement for individual informed consent due to the retrospective study design.

### Study population

For data extraction from the MIMIC-IV database, we used PostgreSQL (v13.7.2) and Navicat Premium (v16) to execute structured queries. The study population consisted of all adult patients (age ≥18 years) in the database admitted to the ICU with a diagnosis of AMI, identified using ICD-9 code 410 and ICD-10 codes I21 and I22. To ensure data completeness and analytical reliability, we applied the following exclusion criteria: (1) patients who died within 24 h of ICU admission, as their clinical records might be incomplete; (2) those with multiple ICU admissions, where only the first ICU admission for AMI was included to avoid data duplication; (3) patients lacking key laboratory measurements obtained on the first day of ICU admission, which were essential for the analyses.

### Variable extraction and processing

Data extraction was performed on the PostgreSQL (v13.7.2) and Navicat Premium (v16.0) platforms using structured query language (SQL). Extracted variables were grouped into six categories: (1) demographic characteristics, among them, race is divided into yes and no based on whether they are white or not, (2) comorbidities, (3) vital signs, (4) laboratory parameters, (5) illness severity scores, and (6) administered treatments. For variables with missing data (23), imputation was performed only if the missing rate was below 30%. We used the “mice” package (v3.16.0) in R, employing a random forest model to conduct multiple imputation. The imputation model included all variables relevant to the primary analysis: the continuous variables to be imputed, completely observed exposure variables (RDW, lactate, total bilirubin, anion gap, urea nitrogen, LDH, and albumin), the completely observed outcome variable (28-day ICU mortality), and other complete categorical and continuous covariates.

### Definition of exposure and clinical outcome

Drawing on previous literature, this study selected six albumin-derived composite indices to comprehensively assess inflammatory activation, nutritional status, and metabolic stress: Red cell distribution width to Albumin Ratio (RAR), Lactate to Albumin Ratio (LAR), Anion Gap to Albumin Ratio (AGAR), Total bilirubin to Albumin Ratio (TAR), Urea nitrogen to Albumin Ratio (UAR), and Lactate Dehydrogenase to Albumin Ratio (LDAR) (16–21). These indices have been previously shown to be associated with adverse outcomes in AMI (16–21). To meet statistical assumptions and enhance clinical interpretability, given that the numerical range of LDAR is notably higher than that of the other five indicators, we applied a logarithmic transformation to LDAR for all subsequent analyses, and LDAR is used to denote the transformed measure throughout the following analyses. The primary clinical endpoint was defined as all-cause death within 28 days of ICU admission for AMI.

### Correlation analysis of albumin-derived biomarkers with 28-day ICU mortality in AMI

Following preliminary hypothesis testing, this study used Cox proportional hazards regression models to analyze the association between the six albumin-derived biomarkers and the clinical endpoint. To control for potential confounding factors, three progressively adjusted regression models were constructed sequentially: Model 1 was an unadjusted crude model; Model 2 adjusted for demographic characteristics (age, sex, race, weight) and major comorbidities; Model 3 further incorporated clinically relevant variables that showed significant differences between survivors and non-survivors, including illness severity scores, key laboratory indicators, and treatment measures. To ensure model stability, multicollinearity diagnosis was performed for all variables included in Model 3 using the Variance Inflation Factor (VIF), and variables with a VIF > 5 were removed. Finally, to explore potential non-linear relationships between key indices (e.g., RAR) and the endpoint, restricted cubic splines (RCS) were fitted, with knots placed at the 5th, 35th, 65th, and 95th percentiles.

### Comparison of predictive performance of albumin-derived biomarkers

The predictive performance of each biomarker for the adverse outcome was assessed by plotting Receiver Operating Characteristic (ROC) curves and calculating the Area under the Curve (AUC). Delong’s test was used to compare the differences in predictive ability among the albumin-derived biomarkers to determine if improvements were statistically significant.

### Subgroup and interaction analysis

To verify the stability of the relationship between the best-performing albumin-derived biomarker (LDAR) and the adverse outcome, prespecified subgroup analyses and interaction tests were conducted. Subgroups were defined based on key characteristics such as age, sex, race, and baseline comorbidities. The consistency of the LDAR effect across subgroups was assessed by testing the statistical significance of interaction terms (*p* for interaction). A non-significant interaction (typically *p* > 0.05) would suggest stability across different populations, while a significant interaction would indicate heterogeneity. All analyses were performed according to a predefined protocol to control bias from multiple comparisons.

### Incremental value of LDAR

LDAR was incorporated into five traditional critical illness scoring systems to construct multivariable Cox proportional hazards models. A composite risk score was calculated for each model using the formula (*β*₁ × variable₁) + (β₂ × variable₂), based on the regression coefficients. Similarly, the predictive performance of each model for the adverse outcome was evaluated by plotting ROC curves and calculating the AUC. Delong’s test was used to compare the predictive ability of models before and after adding LDAR to determine if the improvement was statistically significant.

### Screening of important prognostic features

Within the internal cohort, we first randomly split the data into a training set and a validation set in a 7:3 ratio. During the training phase, four machine learning algorithms were jointly applied to screen for serum laboratory variables closely associated with ICU mortality: the Boruta algorithm (confidence level *p* < 0.01, maximum iterations = 100, with Bonferroni correction), Random Forest (100 trees, maximum depth = 3), Lasso regression (LogLambda min = −5.243), and Gradient Boosting Machine based on the Cox loss function (learning rate = 0.1, boosting rounds = 100). Variables identified as important by all four algorithms were ultimately determined to be the core prognostic features for this cohort.

### Risk prediction modeling and validation

The internal cohort (MIMIC-IV, *n* = 4,210) was randomly split into a training set (70%, *n* = 2,947) and an internal validation set (30%, *n* = 1,263). Feature selection using the four machine learning algorithms (Boruta, Random Forest, Gradient Boosting Machine, and Lasso regression) was performed exclusively on the training set. The seven variables selected by all four algorithms (LDAR, urea nitrogen, lactate, alkaline phosphatase, RDW, glucose, and total bilirubin) were used to build a multivariable Cox proportional hazards regression model. The regression coefficients (*β*) estimated from the training set were fixed to create a locked prediction model. This locked model was then applied without any modification to the internal validation set and the external validation cohort (*n* = 640) to evaluate its discriminative ability (AUC) and calibration (calibration plots and Brier score). All analyses followed the TRIPOD (Transparent Reporting of a Multivariable Prediction Model for Individual Prognosis or Diagnosis) guidelines.

### Statistical analysis

Continuous variables are presented as mean ± standard deviation. Comparisons between groups were performed using Student’s *t*-test or Analysis of Variance (ANOVA), depending on data normality and homogeneity of variance. Categorical variables are presented as numbers (percentages), with comparisons between groups made using Pearson’s chi-square test or Fisher’s exact test, as appropriate. All statistical analyses were performed using R software (version 4.5.1). A two-sided *p*-value < 0.05 was considered statistically significant.

## Results

### Baseline characteristics of the included patients

According to the study’s inclusion criteria, a total of 4,210 critically ill AMI patients were included in the analysis. Baseline characteristics stratified by survival status are presented in Table 1. Compared to survivors, non-survivors were older (74.7 ± 11.2 years vs. 70.9 ± 11.9 years) and had a higher burden of comorbidities, including a greater prevalence of acute kidney injury (AKI), chronic kidney disease (CKD), and heart failure. Illness severity scores (SOFA, APS III, SAPS II, OASIS, APACHE II) were all significantly higher in non-survivors. Regarding vital signs, non-survivors had a higher heart rate, faster respiratory rate, and lower oxygen saturation. Laboratory tests revealed a general deterioration in inflammatory and metabolic markers among non-survivors: levels of RDW, white blood cell count (WBC), lactate, blood glucose, anion gap, potassium, and phosphorus were higher, while albumin, calcium, hemoglobin, red blood cell count, and chloride were lower. Coagulation, liver, and kidney functions were also significantly impaired, as evidenced by prolonged INR, PT, and PTT, and elevated levels of ALT, AST, total bilirubin, creatinine, urea nitrogen, LDH, and ALP. In terms of treatment interventions, non-survivors had a significantly higher rate of continuous renal replacement therapy (CRRT) (24.7% vs. 7.45%), and more frequent use of vasopressors and glucocorticoids, but a lower proportion received antihypertensive therapy. Although the proportion receiving mechanical ventilation was slightly lower, the incidence of shock was higher. Regarding albumin-derived composite indices, all values were significantly higher in the non-survivor group: RAR (6.02 vs. 4.98), AGAR (6.05 vs. 4.83), LAR (1.11 vs. 0.76), UAR (16.1 vs. 10.3), TAR (0.64 vs. 0.36), LDAR (156 vs. 322), and log2 (LDAR) (6.79 vs. 7.54) (all *p* < 0.001). In summary, non-survivors exhibited greater illness severity, worse physiological reserve, and more intensive treatment requirements at baseline, all closely associated with adverse outcomes.

**Table 1:** Baseline characteristics of critically ill AMI patients in the internal derivation cohort, stratified by 28-day ICU survival status.

Variable	Unit	All (*N* = 4,210)	Survivor (*N* = 3,396)	Non-survivor (*N* = 814)	*p* value
Demographics					
Age	Years	71.6 (11.9)	70.9 (11.9)	74.7 (11.2)	<0.001
Gender (Male)	*n* (%)	2,801 (66.5%)	2,254 (66.4%)	547 (67.2%)	0.684
Race (White)	*n* (%)	2,691 (63.9%)	2,203 (64.9%)	488 (60.0%)	0.010
Weight	kg	84.7 (22.5)	85.1 (22.4)	82.8 (22.8)	0.009
Comorbidities					
HTN	*n* (%)	1,531 (36.4%)	1,272 (37.5%)	259 (31.8%)	0.003
AKI	*n* (%)	2,365 (56.2%)	1732 (51.0%)	633 (77.8%)	<0.001
CKD	*n* (%)	1,313 (31.2%)	1,021 (30.1%)	292 (35.9%)	0.002
DM	*n* (%)	1736 (41.2%)	1,409 (41.5%)	327 (40.2%)	0.518
HLD	*n* (%)	2,263 (53.8%)	1873 (55.2%)	390 (47.9%)	<0.001
HF	*n* (%)	2,295 (54.5%)	1818 (53.5%)	477 (58.6%)	0.010
Severity scores					
SOFA	Score	6.36 (3.60)	5.98 (3.42)	7.96 (3.88)	<0.001
APS III	Score	51.7 (21.6)	48.5 (19.8)	65.0 (23.7)	<0.001
SAPS II	Score	42.9 (13.8)	41.0 (12.8)	50.9 (14.7)	<0.001
OASIS	Score	34.3 (8.61)	33.3 (8.20)	38.4 (9.02)	<0.001
APACHE II	Score	21.0 (7.31)	20.1 (6.99)	24.5 (7.61)	<0.001
Vital signs					
HR	Beats/min	88.0 (20.1)	87.0 (19.7)	92.0 (21.0)	<0.001
RR	insp/min	19.3 (6.28)	18.9 (6.23)	21.0 (6.24)	<0.001
NBPS	mmHg	119 (24.9)	119 (24.7)	118 (25.7)	0.271
NBPD	mmHg	67.5 (18.6)	67.6 (18.4)	67.4 (19.3)	0.791
NBPM	mmHg	78.0 [68.0;91.0]	79.0 [68.0;90.0]	78.0 [67.0;91.0]	0.837
SpO₂	%	96.8 (14.1)	97.1 (15.4)	95.7 (5.24)	<0.001
Laboratory parameters					
HCT	%	32.1 (7.08)	32.2 (7.02)	31.7 (7.30)	0.128
Hb	g/dL	10.5 (2.37)	10.5 (2.36)	10.2 (2.41)	0.003
PLT	K/μL	197 (102)	196 (97.3)	198 (118)	0.700
RDW	%	15.2 (2.37)	14.9 (2.19)	16.1 (2.82)	<0.001
RBC	m/μL	3.51 (0.81)	3.53 (0.81)	3.44 (0.83)	0.006
WBC	K/μL	13.6 (8.33)	13.3 (7.62)	14.9 (10.7)	<0.001
ALB	g/dL	3.08 (0.59)	3.14 (0.58)	2.84 (0.61)	<0.001
AG	mEq/L	15.0 (4.75)	14.6 (4.57)	16.5 (5.17)	<0.001
Ca	mg/dL	8.32 (0.81)	8.33 (0.78)	8.26 (0.90)	0.032
Cl	mEq/L	104 (6.76)	104 (6.59)	102 (7.29)	<0.001
Glu	mg/dL	163 (87.9)	159 (85.8)	179 (94.7)	<0.001
K	mEq/L	4.35 (0.79)	4.34 (0.77)	4.43 (0.84)	0.002
Na	mEq/L	138 (5.36)	138 (5.13)	138 (6.23)	0.611
Lac	mmol/L	1.80 [1.30;2.80]	1.80 [1.20;2.62]	2.10 [1.40;3.58]	<0.001
PCO₂	mmHg	42.4 (10.7)	42.1 (10.1)	43.4 (12.8)	0.008
pH	pH units	7.36 (0.10)	7.36 (0.09)	7.33 (0.11)	<0.001
PO₂	mmHg	158 (132)	169 (136)	113 (104)	<0.001
INR	Ratio	1.58 (0.94)	1.54 (0.89)	1.76 (1.11)	<0.001
PT	Seconds	17.2 (9.57)	16.7 (8.85)	19.1 (11.9)	<0.001
PTT	Seconds	44.7 (31.0)	43.8 (30.3)	48.1 (33.7)	0.001
ALT	IU/L	28.0 [16.0;66.0]	27.0 [15.0;59.0]	40.0 [20.0;112]	<0.001
AST	IU/L	46.0 [26.0;114]	42.0 [26.0;97.0]	68.5 [33.0;200]	<0.001
TB	mg/dL	0.60 [0.40;1.10]	0.60 [0.40;1.00]	0.80 [0.40;1.40]	<0.001
CRE	mg/dL	1.76 (1.64)	1.67 (1.59)	2.13 (1.81)	<0.001
URE	mg/dL	33.1 (24.9)	30.6 (22.8)	43.6 (30.1)	<0.001
LDH	U/L	317 [238;481]	301 [230;435]	436 [293;717]	<0.001
ALP	U/L	78.0 [58.0;113]	75.0 [56.0;107]	91.5 [67.0;136]	<0.001
Mg	mg/dL	2.12 (0.53)	2.12 (0.53)	2.11 (0.55)	0.646
PHOS	mg/dL	3.99 (1.50)	3.87 (1.41)	4.49 (1.74)	<0.001
Albumin-derived indices					
RAR	Ratio	5.18 (1.62)	4.98 (1.44)	6.02 (2.04)	<0.001
AGAR	Ratio	5.06 (2.02)	4.83 (1.88)	6.05 (2.29)	<0.001
LAR	Ratio	0.61 [0.40;0.95]	0.57 [0.39;0.89]	0.76 [0.50;1.31]	<0.001
UAR	Ratio	11.4 (9.64)	10.3 (8.61)	16.1 (12.0)	<0.001
TAR	Ratio	0.21 [0.13;0.37]	0.20 [0.13;0.34]	0.26 [0.16;0.52]	<0.001
LDAR	Ratio	106 [75.8;168]	97.9 [72.6;149]	158 [104;258]	<0.001
Log₂(LDAR)	Ratio	6.94 (1.06)	6.79 (0.96)	7.54 (1.24)	<0.001
Treatments					
CRRT	*n* (%)	454 (10.8%)	253 (7.45%)	201 (24.7%)	<0.001
Ventilation	*n* (%)	3,830 (91.0%)	3,105 (91.4%)	725 (89.1%)	0.041
SA	*n* (%)	3,265 (77.6%)	2,584 (76.1%)	681 (83.7%)	<0.001
VP	*n* (%)	3,280 (77.9%)	2,574 (75.8%)	706 (86.7%)	<0.001
GC	*n* (%)	1,168 (27.7%)	851 (25.1%)	317 (38.9%)	<0.001
AHT	*n* (%)	3,723 (88.4%)	3,068 (90.3%)	655 (80.5%)	<0.001

HTN, Hypertension; AKI, Acute Kidney Injury; CKD, Chronic Kidney Disease; DM, Diabetes Mellitus; HLD, Hyperlipidemia; HF, Heart Failure; SOFA, Sequential Organ Failure Assessment; APS III, Acute Physiology Score III; SAPS II, Simplified Acute Physiology Score II; OASIS, Oxford Acute Severity of Illness Score; APACHE II, Acute Physiology and Chronic Health Evaluation II; HR, Heart Rate; NBPS, Non-Invasive Blood Pressure (Systolic); NBPD, Non-Invasive Blood Pressure (Diastolic); NBPM, Non-Invasive Blood Pressure (Mean); RR, Respiratory Rate; SpO₂, Oxygen Saturation; HCT, Hematocrit; Hb, Hemoglobin; PLT, Platelet Count; RDW, Red Cell Distribution Width; RBC, Red Blood Cell Count; WBC, White Blood Cell Count; ALB, Albumin; AG, Anion Gap; Ca, Calcium; Cl, Chloride; Glu, Glucose; K, Potassium; Na, Sodium; Lac, Lactate; PCO₂, Partial Pressure of Carbon Dioxide; pH, Potential of Hydrogen; PO₂, Partial Pressure of Oxygen; INR, International Normalized Ratio; PT, Prothrombin Time; PTT, Partial Thromboplastin Time; ALT, Alanine Aminotransferase; AST, Aspartate Aminotransferase; TB, Total Bilirubin; CRE, Creatinine; URE, Urea Nitrogen; LDH, Lactate Dehydrogenase; ALP, Alkaline Phosphatase; Mg, Magnesium; PHOS, Phosphorus; RAR, RDW to Albumin Ratio; AGAR, Anion Gap to Albumin Ratio; LAR, Lactate to Albumin Ratio; UAR, Urea Nitrogen to Albumin Ratio; TAR, Total Bilirubin to Albumin Ratio; LDAR, Lactate Dehydrogenase to Albumin Ratio; Log₂(LDAR), Log₂-transformed Lactate Dehydrogenase to Albumin Ratio; CRRT, Continuous Renal Replacement Therapy; SA, Sedative Administration; VP, Vasopressor; GC, Glucocorticoids; AHT, Antihypertensive Therapy.

### Association between albumin-derived composite indices and 28-day ICU mortality in AMI patients

The results of the Cox regression analyses are shown in Table 2. In the unadjusted Model 1, all indices showed a significant positive association with mortality risk: RAR (HR = 1.20, 95% CI: 1.17–1.24, *p* < 0.001), AGAR (HR = 1.13, 95% CI: 1.10–1.15, *p* < 0.001), LAR (HR = 1.20, 95% CI: 1.14–1.27, *p* < 0.001), TAR (HR = 1.13, 95% CI: 1.09–1.18, *p* < 0.001), UAR (HR = 1.03, 95% CI: 1.02–1.03, *p* < 0.001), and LDAR (HR = 1.32, 95% CI: 1.25–1.39, *p* < 0.001). These associations remained significant after adjusting for demographics and comorbidities in Model 2. However, in the fully adjusted Model 3, which accounted for inter-group differences, only RAR (HR = 1.14, 95% CI: 1.10–1.19, *p* < 0.001), UAR (HR = 1.01, 95% CI: 1.00–1.02, *p* = 0.012), and LDAR (HR = 1.39, 95% CI: 1.29–1.50, *p* < 0.001) remained independently associated with ICU mortality risk. The associations for AGAR, LAR, and TAR were no longer significant. These results indicate that RAR, UAR, and LDAR have independent predictive value for mortality risk in critically ill patients, with log-transformed LDAR demonstrating a particularly strong association after multivariable adjustment.

**Table 2:** COX regression results of six albumin-derived indices with short-term prognosis in acute myocardial infarction.

Characteristic	Model 1			Model 2			Model 3		
	HR	95% CI	*p*-value	HR	95% CI	*p*-value	HR	95% CI	*p*-value
RAR	1.20	1.17, 1.24	<0.001	1.19	1.15, 1.23	<0.001	1.14	1.10, 1.19	<0.001
AGAR	1.13	1.10, 1.15	<0.001	1.12	1.09, 1.15	<0.001	1.02	0.98, 1.06	0.4
LAR	1.20	1.14, 1.27	<0.001	1.19	1.13, 1.26	<0.001	1.02	0.92, 1.12	0.8
TAR	1.13	1.09, 1.18	<0.001	1.14	1.09, 1.20	<0.001	1.00	0.94, 1.06	>0.9
UAR	1.03	1.02, 1.03	<0.001	1.02	1.02, 1.03	<0.001	1.01	1.00, 1.02	0.012
LDAR	1.32	1.25, 1.39	<0.001	1.34	1.26, 1.41	<0.001	1.39	1.29, 1.50	<0.001

CI, Confidence Interval; HR, Hazard Ratio.

### Non-linear relationships between albumin-derived indices and 28-day ICU mortality

**Figure 1:**
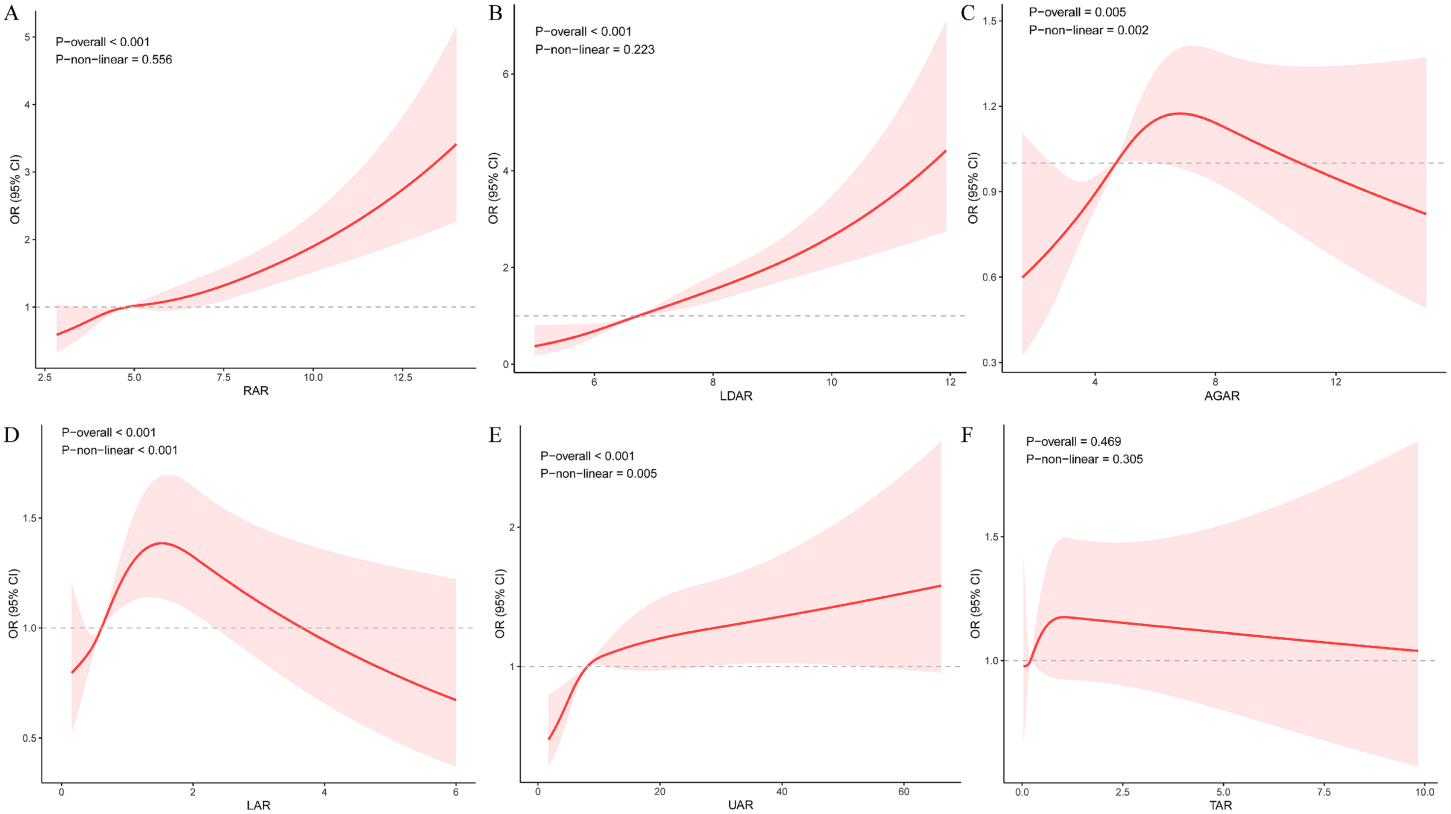
Restricted cubic spline analysis of the dose–response relationship between six albumin-derived composite indices and 28-day ICU mortality. Multivariable-adjusted Cox proportional hazards regression models were used to plot restricted cubic splines in the internal cohort (*N* = 4,210). Knots were placed at the 5th, 35th, 65th, and 95th percentiles. Solid lines represent hazard ratios (HR), and shaded areas indicate 95% confidence intervals. *p*-overall indicates the significance of the overall association, and P-non-linear indicates the significance of the non-linear component. **(A)** RAR (RDW to albumin ratio); **(B)** LDAR (lactate dehydrogenase to albumin ratio); **(C)** AGAR (anion gap to albumin ratio); **(D)** LAR (lactate to albumin ratio); **(E)** UAR (urea nitrogen to albumin ratio); **(F)** TAR (total bilirubin to albumin ratio).

Restricted cubic spline analysis based on the multivariable-adjusted model revealed distinct patterns in the non-linear associations between the albumin-derived indices and mortality risk (Figure 1). RAR showed an approximately linear positive association with mortality risk (*p*-overall < 0.001, *p*-non-linear = 0.556) (Figure 1A), as did LDAR (*p*-overall < 0.001, *p*-non-linear = 0.223) (Figure 1B). In contrast, AGAR, LAR, and UAR all exhibited significant non-linear relationships (all *p*-non-linear < 0.05; Figures 1C–E), suggesting more complex dose–response relationships with potential threshold effects or inflection points. TAR showed no significant association after multivariable adjustment (*p*-overall = 0.469; Figure 1F). These findings indicate that the prognostic impact of some albumin-derived indices (e.g., AGAR, LAR, UAR) is not simply linear, suggesting that their potential non-linear effects should be considered in clinical risk stratification or predictive modeling to improve accuracy and interpretability.

### Comparison of the predictive performance of albumin-derived indices

**Figure 2:**
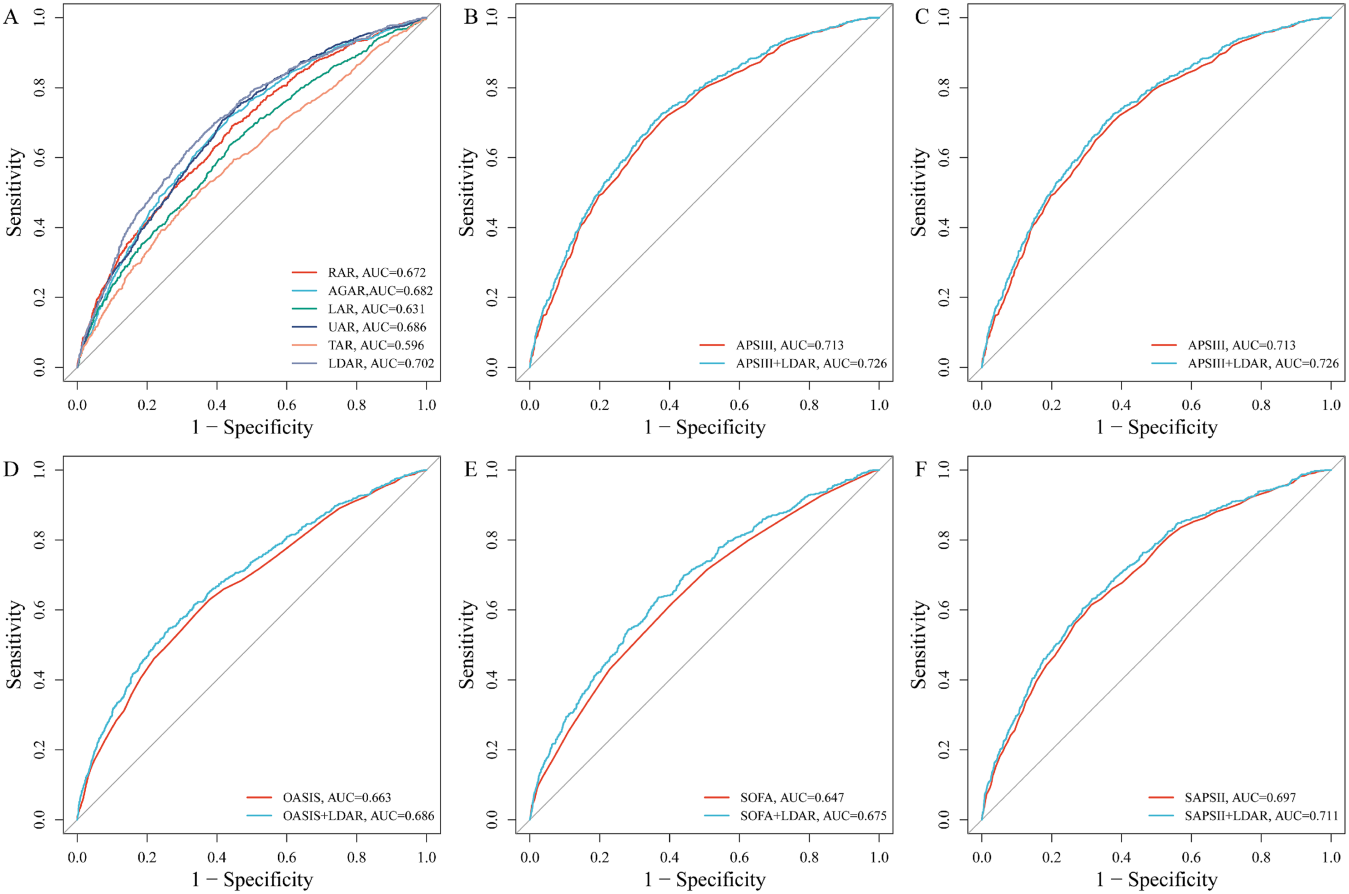
Comparison of predictive performance of six albumin-derived indices for 28-day ICU mortality and incremental value of LDAR to traditional severity scores. **(A)** Receiver operating characteristic (ROC) curves for the six indices; area under the curve (AUC) values are presented as mean (95% confidence interval). DeLong’s test *p* values for pairwise comparisons were all <0.05. **(B–F)** ROC curves for traditional critical illness scores (APACHE II, APS III, OASIS, SOFA, SAPS II) alone and combined with LDAR. DeLong’s test showed significant improvements in AUC after adding LDAR (all *p* < 0.05).

ROC curve analysis revealed significant differences in the discriminatory ability of the various indices for ICU mortality risk (all Delong’s test *p* < 0.05; Figure 2A). LDAR demonstrated the highest predictive performance (AUC = 0.702), followed by UAR (AUC = 0.686), AGAR (AUC = 0.682), and RAR (AUC = 0.672). LAR (AUC = 0.631) and TAR (AUC = 0.596) showed relatively lower predictive ability. These results suggest that indices such as LDAR, UAR, and AGAR possess good discriminatory value for assessing prognosis in critically ill patients and may aid clinical risk stratification. Given its superior performance, LDAR was selected for subsequent focused analysis.

### Subgroup and interaction analysis for LDAR

**Figure 3:**
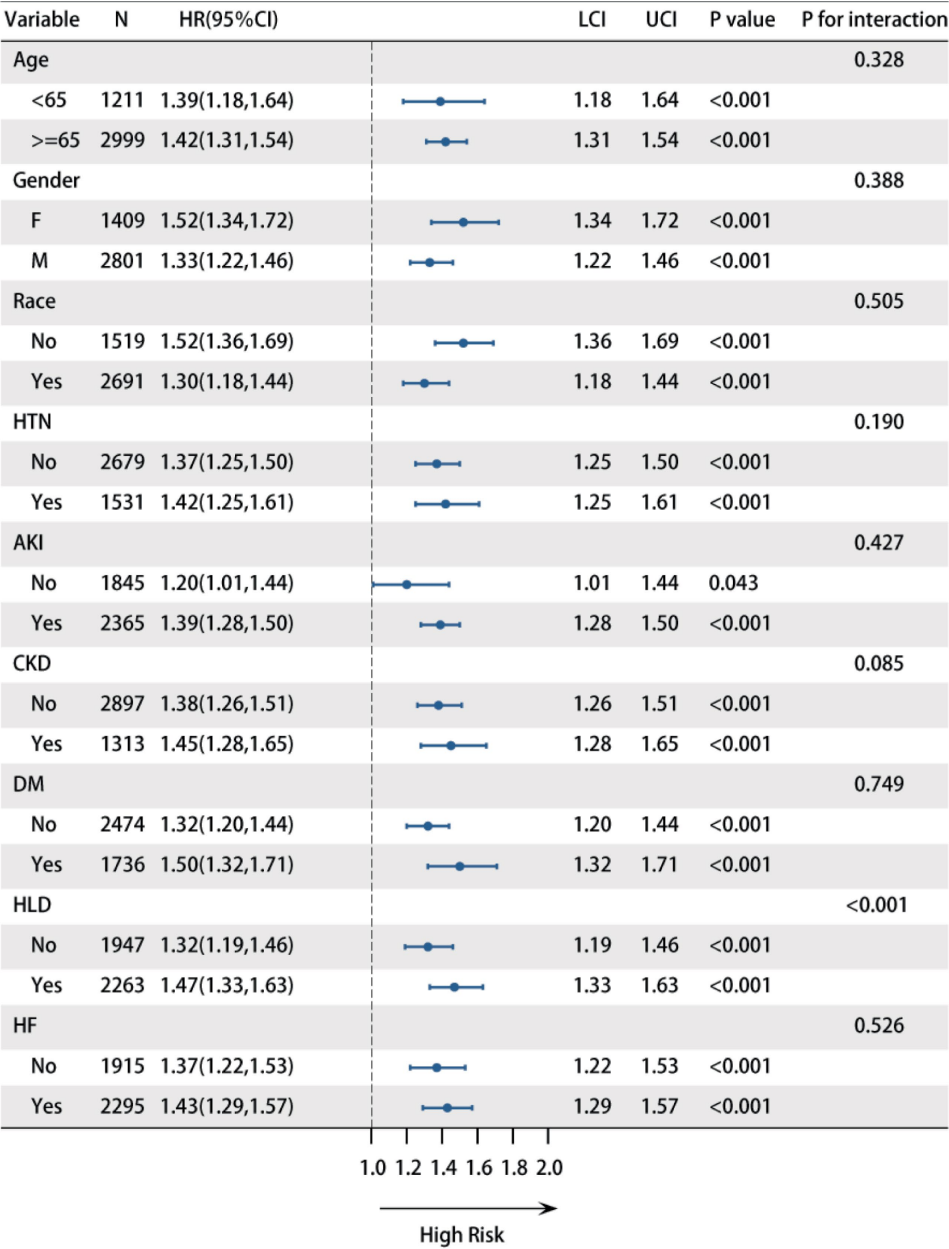
Subgroup analysis and interaction tests for the association between LDAR and 28-day ICU mortality. Multivariable-adjusted Cox regression models were used to assess the effect of LDAR across different demographic and comorbidity subgroups. Squares represent hazard ratios (HR), and horizontal lines indicate 95% confidence intervals. *p* values for interaction reflect the heterogeneity of the LDAR effect between subgroups.

Subgroup analysis and interaction tests were conducted to explore the influence of demographic characteristics and comorbidities on the association between LDAR and ICU mortality risk. After multivariable adjustment, LDAR remained significantly positively associated with mortality risk in the vast majority of subgroups (including different ages, sexes, races, and those with or without hypertension, AKI, CKD, diabetes, and heart failure) (all *p* < 0.05), indicating robust predictive utility (Figure 3). However, interaction testing revealed hyperlipidemia as an important effect modifier. A significant interaction was found between LDAR and hyperlipidemia (*p* for interaction < 0.001). Specifically, the predictive effect of LDAR on mortality risk was stronger in patients with hyperlipidemia (HR = 1.47, 95% CI: 1.33–1.63) compared to those without (HR = 1.32, 95% CI: 1.19–1.46). This suggests that hyperlipidemia may amplify the adverse prognostic risk associated with the metabolic-inflammatory dysregulation and cellular injury reflected by elevated LDAR. No significant interactions were found between LDAR and any other tested variables (e.g., age, sex, race, other comorbidities) (all *p* for interaction > 0.05). In summary, LDAR is a strong predictor of mortality risk in critically ill ICU patients, with an effect that is generally consistent across different populations but significantly modified by the presence of hyperlipidemia. This highlights the potential for enhanced risk stratification using LDAR, particularly in patients with hyperlipidemia.

### Incremental value of LDAR

**Figure 4:**
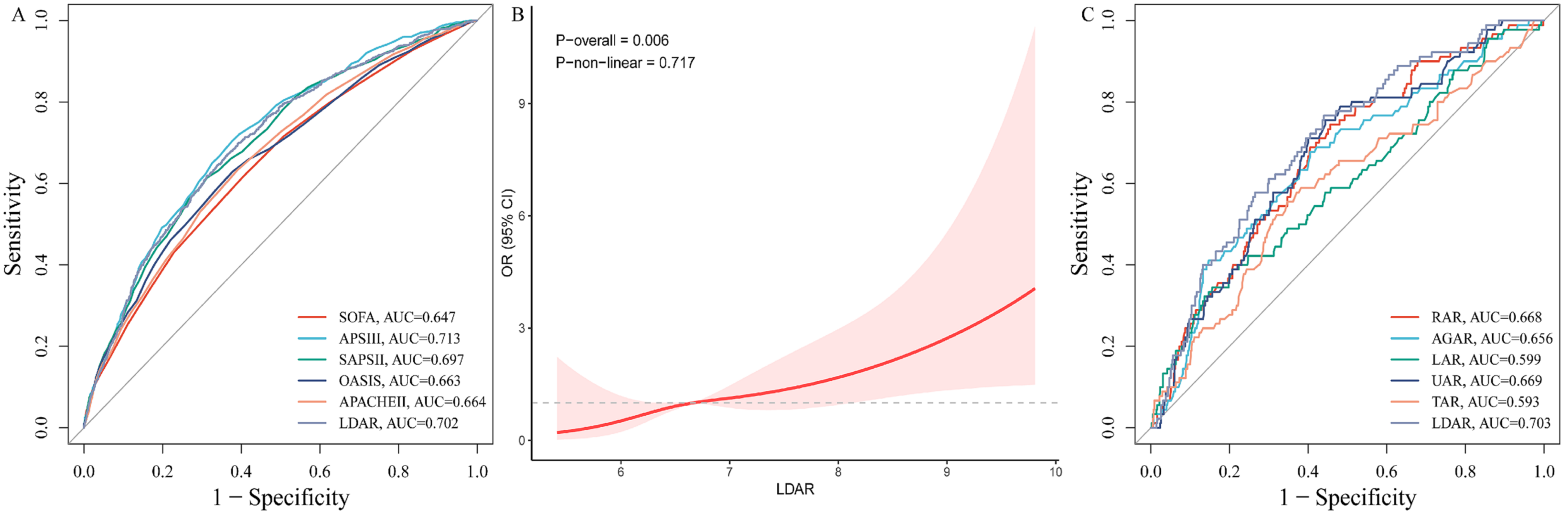
Validation of LDAR’s predictive performance in internal and external cohorts. **(A)** Bar chart comparing AUC values of LDAR with traditional severity scores in the internal cohort. **(B)** Dose–response relationship between LDAR and 28-day ICU mortality in the external validation cohort (*N* = 640) using restricted cubic spline analysis (multivariable-adjusted). **(C)** Comparison of AUC values for six albumin-derived indices in predicting 28-day ICU mortality in the external cohort, demonstrating LDAR remained the best performer (AUC = 0.703).

To assess the incremental predictive value of LDAR for ICU mortality risk, we combined it with five established critical illness severity scoring systems (APACHE II, APS III, OASIS, SOFA, and SAPS II). The results showed that incorporating LDAR consistently improved the predictive accuracy of all traditional models (all Delong’s test *p* < 0.05). Specifically, the AUC for APACHE II increased from 0.664 to 0.685 (Figure 2B); APS III from 0.713 to 0.726 (Figure 2C); OASIS from 0.663 to 0.686 (Figure 2D); SOFA from 0.647 to 0.675 (Figure 2E); and SAPS II from 0.697 to 0.711 (Figure 2F). Notably, the predictive ability of LDAR alone (AUC = 0.702) was already superior to the SOFA (0.647), APACHE II (0.664), and OASIS (0.663) scores, and comparable to SAPS II (0.697) and APS III (0.713) (Figure 4A). These results indicate that LDAR not only provides incremental prognostic information to traditional scoring systems but also possesses good independent discriminatory power, even outperforming some established scores.

### External validation in a real-world cohort

The external validation cohort included 640 critically ill AMI patients (28-day mortality = 14.06%) (Supplementary Table S1). In a Cox regression model adjusted for all potential covariates, a higher LDAR level was significantly associated with an increased risk of 28-day mortality (HR = 1.63; 95% CI: 1.24–2.13, *p* < 0.001). Subsequent RCS analysis confirmed a significant linear positive dose–response relationship between LDAR and short-term ICU mortality in this external cohort (Figure 4B). Furthermore, even in the external cohort, the predictive ability of LDAR for mortality risk (AUC = 0.703) remained superior to the other five albumin-derived indices: RAR (0.668), AGAR (0.656), LAR (0.609), UAR (0.667), and TAR (0.593) (Figure 4C).

### Optimal cutoff value of LDAR for predicting 28-day ICU mortality and its validation in internal and external cohorts

To enhance the clinical utility of the LDAR, the optimal cutoff value of log-transformed LDAR for predicting 28-day ICU mortality was determined in the derivation cohort (MIMIC-IV, *n* = 4,210) based on the receiver operating characteristic (ROC) curve by maximizing the Youden index. The optimal cutoff was identified as 6.850, corresponding to a sensitivity of 69.8%, a specificity of 60.9%, and a Youden index of 0.307. This finding indicates that, using a cutoff of 6.850, LDAR identifies approximately 70% of patients at risk of death while correctly ruling out approximately 61% of non-survivors. In the external validation cohort (ICU of the Affiliated Hospital of Guizhou Medical University, *n* = 640), the same cutoff value (6.850) yielded a sensitivity of 66.7%, a specificity of 63.3%, and a Youden index of 0.299. The performance metrics were closely aligned between the internal and external cohorts, suggesting that this cutoff value exhibits satisfactory stability and generalizability across different populations, further supporting the reliability of LDAR as a prognostic marker.

### Development and validation of a risk prediction model for high-risk AMI patients

**Figure 5:**
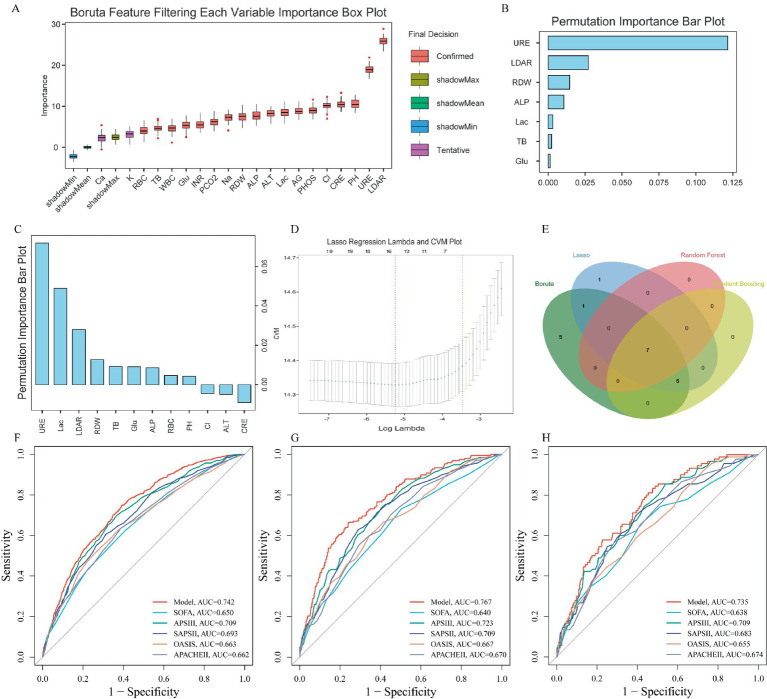
Machine learning-based selection of key prognostic variables and development/validation of a risk prediction model. **(A–D)** Four machine learning algorithms (Boruta, Random Forest, Gradient Boosting Machine, Lasso regression) were used to identify laboratory variables strongly associated with 28-day ICU mortality. **(E)** Venn diagram showing seven core variables commonly selected by all four algorithms: LDAR, urea nitrogen (URE), lactate (Lac), alkaline phosphatase (ALP), RDW, glucose (Glu), and total bilirubin (TB). **(F–H)** ROC curves for the Cox risk model based on these seven variables in the internal training set [**(F)**, AUC = 0.742], internal validation set [**(G)**, AUC = 0.767], and external cohort [**(H)**, AUC = 0.735], with comparisons to traditional severity scores.

Within the internal training cohort, four machine learning algorithms identified the following numbers of variables closely associated with ICU mortality: 18 variables (Boruta algorithm; Figure 5A); 7 variables (Random Forest; Figure 5B); 12 variables (Gradient Boosting; Figure 5C); and 14 variables (Lasso regression; Figure 5D). A Venn diagram subsequently identified seven key variables shared across all four methods: LDAR, urea nitrogen (URE), lactate (Lac), alkaline phosphatase (ALP), RDW, glucose (Glu), and total bilirubin (TB) (Figure 5E). These were used to construct a risk assessment model to predict 28-day mortality in AMI patients. The model formula is as follows: Risk Score = (0.1790963362 × LDAR) + (0.0900971225 × RDW) + (0.0005723322 × Glu) + (0.0346863127 × Lac) + (0.0103747682 × URE) + (0.0052965180 × TB) + (0.0004895633 × ALP). Compared to the five traditional severity scoring systems, this model demonstrated higher sensitivity and specificity for predicting 28-day ICU mortality (Figures 5F–H). The AUC was 0.742 in the internal training cohort (Figure 5F), 0.767 in the internal validation cohort (Figure 5G), and 0.735 in the external real-world cohort (Figure 5H). These consistent results indicate that the model exhibits robust stability and predictive performance across different cohorts. The calibration curves show good agreement between predicted and observed risks across deciles of predicted probability, and the Brier scores indicate acceptable overall calibration.

## Discussion

Cardiovascular disease remains the leading cause of death globally, with AMI being a critical event associated with high mortality (1–4). While reperfusion and pharmacological therapies have improved overall AMI outcomes, the prognosis for critically ill patients, particularly those admitted to the ICU, remains severe. For instance, cardiogenic shock following AMI still carries a 28-day mortality rate of approximately 40%, imposing a substantial burden on patients, families, and society. Consequently, early clinical intervention and accurate prognostic assessment are crucial for reducing mortality and improving long-term outcomes. Although several prognostic scoring systems exist for AMI, their complexity and inconvenience often limit clinical application (5, 6). Developing simple yet accurate biomarkers to facilitate early high-risk patient identification and guide treatment has therefore become an important avenue for improving AMI management (7–9).

In this study, we systematically evaluated the predictive performance of six albumin-derived composite indices for 28-day ICU mortality in critically ill AMI patients. Our results indicate that after adjusting for multiple potential confounders, RAR, UAR, and LDAR were independent predictors of mortality risk. Among these, the logarithmically transformed LDAR demonstrated the strongest discriminatory ability, with an area under the receiver operating characteristic curve (AUC = 0.702) significantly outperforming other indices. Its performance remained robust in an independent external validation cohort (AUC = 0.703). Importantly, LDAR not only performed well alone but also significantly enhanced the discrimination of traditional critical illness scoring systems like APACHE II and SOFA when integrated into them (all DeLong’s test *p* < 0.05). Subgroup analysis further revealed that the predictive effect of LDAR was generally consistent across various demographic and comorbidity subgroups, except for a significant interaction with hyperlipidemia. This suggests its risk stratification value may be particularly pronounced in this specific population. Collectively, this evidence underscores LDAR as a novel biomarker combining convenience and efficacy, with significant potential for early risk stratification in critically ill AMI patients.

Mechanistically, the superior predictive performance of LDAR stems from its unique composition, which captures two key pathophysiological dimensions simultaneously: the extent of acute local tissue injury and the systemic homeostatic reserve. Specifically, this index integrates clinical information from LDH and albumin: LDH is a sensitive marker of cell damage and necrosis, and its level directly reflects the extent and severity of myocardial necrosis post-AMI, representing the intensity of “local injury” (24–26). In contrast, serum albumin level is a composite indicator of nutritional status, systemic inflammation, and hepatic synthetic function. Its decrease (hypoalbuminemia) signifies a collapse of “systemic homeostasis” and decompensation (10–12). In critically ill AMI patients, extensive myocardial necrosis (high LDH) and severe systemic inflammatory consumption or malnutrition (low albumin) often coexist and exacerbate each other, forming a vicious cycle (20, 27, 28). Therefore, an elevated LDAR reflects a high-risk profile of “severe injury coupled with poor reserve”. While this index provides a more comprehensive pathophysiological assessment than single biomarkers, its prognostic discriminatory power is only moderate (AUC ~ 0.70), and it should be interpreted alongside other clinical variables.

Furthermore, a noteworthy finding of this study is the significant prognostic interaction between LDAR and hyperlipidemia. The underlying pathophysiological mechanism may involve a synergistic exacerbation of the “metabolism-inflammation-endothelial injury” vicious cycle. First, hyperlipidemia is often associated with hepatic steatosis and systemic oxidative stress, which impair hepatic functional reserve. This makes albumin synthesis more susceptible to suppression and its consumption more pronounced during inflammatory states. Concurrently, chronic endothelial cell stress in hyperlipidemia may render it more vulnerable to injury and death following acute insults like ischemia, leading to a more significant release of lactate dehydrogenase (29–31). Second, the endothelial dysfunction caused by hyperlipidemia and the compromised endothelial protection resulting from hypoalbuminemia likely act additively, predisposing the vascular system to diffuse injury and microcirculatory disturbance under critical illness, thereby worsening organ hypoperfusion (32, 33). Finally, a liver with pre-existing steatosis is more prone to acute dysfunction under stress, manifesting as a precipitous drop in albumin synthesis and a decline in LDH clearance, both driving the LDAR level upward (34). In summary, hyperlipidemia provides a “frail” metabolic and vascular substrate, while LDAR, as a composite index, simultaneously reflects the amplified cell injury from the underlying condition and the acute collapse of synthetic function, thus exhibiting stronger predictive power for mortality risk in patients with concomitant hyperlipidemia.

Compared to previous studies, our research possesses several strengths in design and validation. First, in terms of design, we conducted a systematic head-to-head comparison of six common albumin-derived indices, rather than validating a single marker. The dual-cohort analysis clearly established the superior relative performance of LDAR, providing direct evidence for clinical biomarker selection. Second, regarding depth, we not only validated the independent and incremental predictive value of LDAR but also, through subgroup analysis, revealed its interaction with hyperlipidemia. Furthermore, we employed multiple machine learning algorithms to build and validate a multivariate prediction model incorporating LDAR, achieving a progressive development from biomarker identification to a practical risk assessment tool. Additionally, rigorous external validation confirmed the robustness and generalizability of both LDAR and the derived model across different populations, enhancing the reliability of our conclusions. To our knowledge, this is also the first large-cohort study validating the relationship between LDAR, AGAR, TAR, and adverse outcomes in critically ill AMI patients.

Furthermore, from a clinical perspective, an LDAR > 6.850 may serve as a reference threshold for rapid risk stratification in critically ill patients with AMI within 24 h of admission: patients with values exceeding this cutoff exhibit a significantly higher 28-day mortality risk and may warrant more intensive monitoring or aggressive interventions. However, it should be noted that both the sensitivity and specificity of this cutoff are moderate (approximately 60–70%), which aligns with the overall discriminative ability of LDAR as a single predictor (AUC ≈ 0.70). Therefore, it is not recommended to rely solely on this threshold for binary decision-making; instead, it should be integrated with other key clinical variables (e.g., blood urea nitrogen, lactate, RDW) as demonstrated in the multivariable machine learning models developed in this study, to achieve more precise risk assessment.

It should be emphasized that hemolysis of blood samples may affect the measurement accuracy of certain laboratory parameters, particularly LDH, potentially introducing bias into the calculation of LDAR. In the MIMIC-IV database, the laboratory data have undergone preliminary quality control; clinical laboratories typically reject or flag hemolyzed specimens as unreliable based on visual inspection, free hemoglobin levels, or hemolysis indices. However, due to the retrospective nature of the database, we cannot directly ascertain the hemolysis status of individual samples. This, therefore, represents an inherent limitation of database studies. Nevertheless, two factors support the reliability of our findings: (1) The consistent predictive performance of LDAR in an external validation cohort (from the Affiliated Hospital of Guizhou Medical University), where hemolyzed samples are routinely rejected and not used for clinical testing, suggests that the observed associations are not merely an artifact of sample hemolysis; and (2) the robustness of the results across two independent cohorts with different laboratory protocols further mitigates this concern.

Despite these meaningful findings, several limitations of this study must be acknowledged. First, due to the inherent limitations of retrospective study designs, residual confounding cannot be completely ruled out. Moreover, although our adjustment strategy was informed by clinical knowledge and prior literature, the initial screening of covariates was based on univariable significance testing between survivors and non-survivors—a pragmatic but suboptimal approach that is nevertheless common in retrospective database studies. This method may lead to data-driven overfitting and does not guarantee proper control of confounders. Future prospective studies with pre-specified causal frameworks are needed to validate our findings. Second, all our analyses were based on laboratory data obtained during the early admission period (within 24 h). We did not capture the dynamic trajectories of these indicators throughout the ICU stay, which may contain important prognostic information. Third, although we used ICD codes to identify AMI patients, a standard method in database research, the possibility of inadvertently including a small number of non-type 1 myocardial infarctions (e.g., type 2) cannot be entirely excluded. However, given the rarity of such cases in critically ill populations and the consistency of our findings across two cohorts, this potential misclassification is unlikely to have substantially influenced the results. Fourth, key clinical variables were unavailable, including troponin levels (a gold-standard marker of myocardial necrosis) and detailed information on revascularization procedures (e.g., PCI or CABG). The absence of these critical data limits the comprehensiveness of our analysis and may have influenced the observed predictive performance. Fifth, although we used an external validation cohort, it was effectively single-center (Affiliated Hospital of Guizhou Medical University). The MIMIC-IV database, despite its large sample size, also originates from a single institution (Beth Israel Deaconess Medical Center). Therefore, our findings have not yet been tested in a truly multi-center or diverse healthcare setting. Consequently, the generalizability of LDAR and the derived model remains to be established.

## Conclusion

In this two-cohort retrospective analysis, LDAR was identified as the most effective among six albumin-derived composite indices for predicting 28-day ICU mortality in critically ill myocardial infarction patients, albeit with moderate discriminative ability (AUC ~ 0.70). LDAR modestly enhanced the discriminatory ability of traditional critical illness scores and showed a significant interaction with hyperlipidemia. A machine learning-based model incorporating LDAR and six other variables achieved acceptable but not robust performance in internal and external validation (AUCs 0.767 and 0.735). Given the moderate predictive accuracy, the absence of key variables (e.g., troponin, revascularization data), and the single-center nature of both cohorts, we caution against overinterpreting these results as ready for clinical implementation. LDAR may serve as a supplementary biomarker for early risk assessment, but its clinical utility and generalizability require rigorous evaluation in multi-center prospective studies before any practical application can be recommended.

## Data Availability

The original contributions presented in the study are included in the article/Supplementary material, further inquiries can be directed to the corresponding author/s.
