# Supplementary material for: LDAR outperforms other albumin-derived indices in predicting 28-day ICU mortality in critically ill myocardial infarction patients: a two-cohort study
Source: Front Med (Lausanne). 2026 May 15;13:1801925. doi: 10.3389/fmed.2026.1801925 (PMC13219332; doi:10.3389/fmed.2026.1801925)
Supplement: Supplementary file 1 [file Supplementary_file_1.docx]

**Table S1. Baseline characteristics of critically ill AMI patients in the external validation cohort, stratified by 28-day ICU survival status**

| **Variable** | **Unit** | **All (N=640)** | **Survivor (N=550)** | **Non-survivor (N=90)** | **P value** |
| --- | --- | --- | --- | --- | --- |
| **Demographics** | | | | | |
| Age | years | 71.8 (11.7) | 71.3 (11.7) | 74.6 (11.5) | 0.014 |
| Gender (Male) | n (%) | 436 (68.1%) | 373 (67.8%) | 63 (70.0%) | 0.772 |
| BMI | kg/m² | 27.6 (4.73) | 27.6 (4.79) | 27.8 (4.36) | 0.679 |
| **Comorbidities** | | | | | |
| HTN | n (%) | 216 (33.8%) | 182 (33.1%) | 34 (37.8%) | 0.452 |
| AKI | n (%) | 352 (55.0%) | 282 (51.3%) | 70 (77.8%) | <0.001 |
| CKD | n (%) | 207 (32.3%) | 179 (32.5%) | 28 (31.1%) | 0.882 |
| DM | n (%) | 268 (41.9%) | 229 (41.6%) | 39 (43.3%) | 0.851 |
| HF | n (%) | 357 (55.8%) | 309 (56.2%) | 48 (53.3%) | 0.697 |
| **Severity scores** | | | | | |
| SOFA | score | 6.36 (3.51) | 6.10 (3.39) | 7.92 (3.86) | <0.001 |
| APS III | score | 51.6 (21.5) | 49.4 (20.4) | 65.2 (23.0) | <0.001 |
| SAPS II | score | 42.6 (13.2) | 41.2 (12.5) | 51.5 (14.0) | <0.001 |
| OASIS | score | 34.4 (8.35) | 33.7 (8.10) | 38.7 (8.59) | <0.001 |
| APACHE II | score | 20.9 (7.06) | 20.3 (6.82) | 24.7 (7.33) | <0.001 |
| **Vital signs** | | | | | |
| HR | beats/min | 86.8 (20.5) | 86.0 (20.1) | 92.0 (22.5) | 0.018 |
| RR | insp/min | 19.6 (6.73) | 19.3 (6.69) | 21.2 (6.83) | 0.017 |
| NBPS | mmHg | 119 (25.2) | 119 (24.8) | 123 (27.4) | 0.247 |
| NBPD | mmHg | 68.4 (19.5) | 68.2 (19.5) | 69.8 (19.5) | 0.472 |
| NBPM | mmHg | 81.6 (19.2) | 81.2 (19.2) | 84.0 (19.0) | 0.198 |
| SpO₂ | % | 96.8 (3.94) | 96.9 (3.62) | 96.1 (5.48) | 0.17 |
| **Laboratory parameters** | | | | | |
| HCT | % | 32.5 (7.32) | 32.7 (7.30) | 31.4 (7.38) | 0.127 |
| Hb | g/dL | 10.6 (2.44) | 10.7 (2.44) | 10.1 (2.43) | 0.05 |
| PLT | K/μL | 195 (97.1) | 196 (96.6) | 188 (101) | 0.444 |
| RDW | % | 15.1 (2.31) | 14.9 (2.15) | 16.3 (2.81) | <0.001 |
| RBC | m/μL | 3.56 (0.83) | 3.59 (0.83) | 3.39 (0.81) | 0.038 |
| WBC | K/μL | 13.7 (8.61) | 13.5 (8.46) | 15.0 (9.40) | 0.154 |
| ALB | g/dL | 3.12 (0.54) | 3.15 (0.54) | 2.97 (0.53) | 0.004 |
| AG | mEq/L | 14.7 (4.68) | 14.4 (4.57) | 16.4 (5.02) | 0.001 |
| Ca | mg/dL | 8.34 (0.78) | 8.33 (0.74) | 8.42 (1.03) | 0.406 |
| Cl | mEq/L | 104 (6.94) | 104 (6.94) | 103 (6.89) | 0.135 |
| Glu | mg/dL | 162 (86.4) | 160 (84.2) | 177 (98.3) | 0.113 |
| K | mEq/L | 4.33 (0.75) | 4.32 (0.75) | 4.39 (0.72) | 0.412 |
| Na | mEq/L | 138 (5.43) | 138 (5.42) | 138 (5.52) | 0.961 |
| Lac | mmol/L | 2.30 (1.79) | 2.20 (1.61) | 2.92 (2.54) | 0.011 |
| PCO₂ | mmHg | 42.5 (10.5) | 42.4 (10.3) | 43.1 (11.9) | 0.591 |
| pH | pH units | 7.36 (0.10) | 7.36 (0.09) | 7.33 (0.10) | 0.006 |
| PO₂ | mmHg | 166 (137) | 172 (138) | 130 (123) | 0.003 |
| INR | ratio | 1.60 (1.09) | 1.53 (0.93) | 2.04 (1.73) | 0.008 |
| PT | seconds | 17.3 (11.2) | 16.5 (9.15) | 22.0 (19.0) | 0.008 |
| PTT | seconds | 43.7 (30.4) | 43.4 (30.3) | 45.6 (31.1) | 0.541 |
| ALT | IU/L | 124 (435) | 131 (466) | 78.0 (125) | 0.027 |
| AST | IU/L | 211 (761) | 219 (816) | 159 (230) | 0.155 |
| TB | mg/dL | 1.16 (2.41) | 1.06 (2.10) | 1.79 (3.76) | 0.075 |
| CRE | mg/dL | 1.67 (1.51) | 1.60 (1.46) | 2.11 (1.73) | 0.008 |
| URE | mg/dL | 31.9 (22.9) | 30.5 (22.5) | 40.3 (24.0) | <0.001 |
| LDH | U/L | 467 (667) | 449 (694) | 580 (450) | 0.02 |
| ALP | U/L | 105 (124) | 102 (106) | 125 (202) | 0.302 |
| Mg | mg/dL | 2.14 (0.50) | 2.15 (0.51) | 2.09 (0.47) | 0.314 |
| **Albumin-derived indices** | | | | | |
| RAR | ratio | 5.03 (1.42) | 4.92 (1.38) | 5.68 (1.48) | <0.001 |
| AGAR | ratio | 4.87 (1.91) | 4.74 (1.88) | 5.67 (1.89) | <0.001 |
| LAR | ratio | 0.77 (0.70) | 0.73 (0.65) | 1.01 (0.91) | 0.006 |
| UAR | ratio | 10.8 (8.84) | 10.3 (8.79) | 14.0 (8.49) | <0.001 |
| TAR | ratio | 0.39 (0.86) | 0.36 (0.79) | 0.60 (1.19) | 0.063 |
| Log₂(LDAR) | ratio | 6.85 (0.94) | 6.77 (0.92) | 7.35 (0.90) | <0.001 |
| **Treatments** | | | | | |
| Ventilation | n (%) | 582 (90.9%) | 509 (92.5%) | 73 (81.1%) | 0.001 |
| SA | n (%) | 506 (79.1%) | 436 (79.3%) | 70 (77.8%) | 0.854 |
| VP | n (%) | 495 (77.3%) | 416 (75.6%) | 79 (87.8%) | 0.016 |

Abbreviations: HTN: Hypertension; AKI: Acute Kidney Injury; CKD: Chronic Kidney Disease; DM: Diabetes Mellitus; HF: Heart Failure; SOFA: Sequential Organ Failure Assessment; APS III: Acute Physiology Score III; SAPS II: Simplified Acute Physiology Score II; OASIS: Oxford Acute Severity of Illness Score; APACHE II: Acute Physiology and Chronic Health Evaluation II; HR: Heart Rate; RR: Respiratory Rate; NBPS: Non-Invasive Blood Pressure (Systolic); NBPD: Non-Invasive Blood Pressure (Diastolic); NBPM: Non-Invasive Blood Pressure (Mean); SpO₂: Oxygen Saturation; HCT: Hematocrit; Hb: Hemoglobin; PLT: Platelet Count; RDW: Red Cell Distribution Width; RBC: Red Blood Cell Count; WBC: White Blood Cell Count; ALB: Albumin; AG: Anion Gap; Ca: Calcium; Cl: Chloride; Glu: Glucose; K: Potassium; Na: Sodium; Lac: Lactate; PCO₂: Partial Pressure of Carbon Dioxide; pH: Potential of Hydrogen; PO₂: Partial Pressure of Oxygen; INR: International Normalized Ratio; PT: Prothrombin Time; PTT: Partial Thromboplastin Time; ALT: Alanine Aminotransferase; AST: Aspartate Aminotransferase; TB: Total Bilirubin; CRE: Creatinine; URE: Urea Nitrogen; LDH: Lactate Dehydrogenase; ALP: Alkaline Phosphatase; Mg: Magnesium; RAR: RDW to Albumin Ratio; AGAR: Anion Gap to Albumin Ratio; LAR: Lactate to Albumin Ratio; UAR: Urea Nitrogen to Albumin Ratio; TAR: Total Bilirubin to Albumin Ratio; Log₂(LDAR): Log₂-transformed Lactate Dehydrogenase to Albumin Ratio; SA: Sedative Administration; VP: Vasopressor.
